# Clinical characteristics and survival prediction of surgical patients with invasive pancreatic cystic neoplasm: a large retrospective study over two decades

**DOI:** 10.1186/s12957-023-03145-z

**Published:** 2023-08-23

**Authors:** Yanjing Song, Zhe Li, Hongyuan Cui, Jingyong Xu, Jinghai Song

**Affiliations:** Department of General Surgery, Department of Hepato-Bilio-Pancreatic Surgery, Beijing Hospital, National Center of Gerontology, Institute of Geriatric Medicine, Chinese Academy of Medical Sciences, NO. 1 DaHua Road, Dong Dan, Beijing, 100730 People’s Republic of China

**Keywords:** Pancreatic cystic neoplasms (PCN), Overall survival (OS), Cancer-specific survival (CSS), Prognostic factors, Nomogram

## Abstract

**Purposes:**

Invasive pancreatic cystic neoplasms (iPCNs) are an uncommon and biologically heterogeneous group of malignant neoplasms. We aimed to investigate the clinicopathological characteristics of iPCN patients and to develop nomograms for individual survival prediction after radical surgery.

**Methods:**

Data of patients diagnosed with iPCN and pancreatic ductal adenocarcinoma (PDAC) between 2000 and 2018 from the SEER database were retrieved. The differences in clinical outcomes were evaluated using the Kaplan–Meier analysis. Nomograms were proposed based on the Cox regression model and internally validated by C-index, area under the curve (AUC) value, and calibration plot.

**Results:**

A total of 7777 iPCN patients and 154,336 PDAC patients were enrolled. Most neoplasms were advanced, with 63.1% at stage IV. The 3-year overall survival (OS) and cancer-specific survival (CSS) rates in surgical patients were as follows: 45.7% and 50.1% for invasive intraductal papillary mucinous neoplasm (IPMN), 54.8% and 59.3% for invasive mucinous cystic neoplasm (MCN), 97.8% and 98.2% for invasive solid pseudopapillary neoplasm (SPN), 88.9% and 88.9% for invasive serous cystic neoplasm (SCN), and 27.3% and 30.5% for PDAC. Subgroup analyses showed no clinical benefit from chemotherapy or radiotherapy in lymph node-negative iPCN patients who underwent surgery. The following variables associated with OS and CSS were identified: age, race, chemotherapy, radiotherapy, histologic type, pathological grade, regional nodes examined, and T, N, and M stage. The nomograms had good discrimination and calibration by internal validation, with an AUC value of 0.800 for 3-year OS and 0.814 for 3-year CSS.

**Conclusion:**

Our study showed that the prognosis of iPCN patients was significantly better than PDAC patients. The proposed nomograms demonstrated substantially better discrimination and calibration.

**Supplementary Information:**

The online version contains supplementary material available at 10.1186/s12957-023-03145-z.

## Introduction

Pancreatic cystic neoplasm (PCN) is a lesion with heterogeneous proliferation of pancreatic epithelial tissue, characterized by unilocular or multilocular cavity formation due to retention of secretion. According to the 2019 WHO classification of pancreatic tumors [1], there are four main types of PCN: intraductal papillary mucinous neoplasm (IPMN), mucinous cystic neoplasm (MCN), serous cystic neoplasm (SCN), solid pseudopapillary neoplasm (SPN), and other rare types, such as cystic neuroendocrine tumors (cNET), most of which encompasses variable grades of lesions ranging from benign neoplasms to invasive cancer (referred to as invasive PCN). Furthermore, PCN can also be categorized into serous or mucinous neoplasms, the prognosis of which is completely different [2].

With the development of medical imaging, the rate of detection of PCN has greatly increased. PCN were detected in up to 49.1% of tested individuals using magnetic resonance cholangiopancreatography [3]. IPMN is the most common PCN, followed by MCN, SCN, and SPN, all of which account for approximately 90% of cystic tumors of the pancreas [4]. The malignant potential varies between PCN types, and the prognosis is diverse. Previous studies suggested that SCNs are generally benign [5], whereas IPMN, MCN, and SPN are precancerous lesions that require long-term monitoring. In several studies, approximately 11–30% of side-branch (SB)-IPMN and 10–39% of MCN developed into advanced neoplasia (high-grade dysplasia or invasive cancer) [2], implying inaccuracies in the choice of operative indication, and more in-depth research on the clinicopathological features of PCN is needed. Currently, reliable survival statistics regarding prognosis and risk factors contributing to differences between types remain uncertain. The American Joint Committee on Cancer (AJCC) TNM staging system, a widely used criterion for staging patients with invasive PCN (iPCN), analogous to pancreatic ductal adenocarcinoma (PDAC), may not meet the need for accurate survival prediction for an individual patient with particular characteristics.

In this retrospective analysis, we comprehensively investigated the clinicopathological characteristics of patients with various iPCN types from the US Surveillance, Epidemiology, and End Results (SEER) database to evaluate survival differences among pathological types, including PDAC. We subsequently created and validated new nomograms for predicting overall survival (OS) and cancer-specific survival (CSS) of surgical iPCN patients based on the identified prognostic factors, and compared the predictive values with the AJCC 8th TNM staging system. Finally, we aimed to offer a clinically practical method to guide physicians in the postoperative management of surgical patients with iPCN.

## Method

### Patients and data collection

Data of patients diagnosed with pancreatic cancer (PC) between 2000 and 2018 from the SEER-18 database were retrieved using the National Cancer Institute’s SEER*Stat software (version 8.3.9.2). According to the ICD-O-3, neoplasms were pathologically confirmed using the following topography and morphology codes: C25.0–C25.9 for PC; 8050/3, 8260/3, 8450/3, 8453/3, 8471/3, 8480/3, 8481/3, and 8503/3 for invasive IPMN (iIPMN), also defined as IPMN with an associated invasive carcinoma; 8440/3 and 8470/3 for invasive MCN (iMCN), also defined as MCN with an associated invasive carcinoma; 8452/3 for invasive SPN (iSPN); 8441/3 and 8460/3 for invasive SCN (iSCN), also defined as serous cystadenocarcinoma; and 8010/3, 8140/3, and 8500/3 for PDAC. In our study, patients with available information on survival time who were diagnosed with iPCN or PDAC by positive histology from 2000 to 2018 were included, and the following patients were excluded when performing statistical analysis: (a) patients who were younger than 19 years or older than 99 years; (b) patients whose postoperative survival time was less than one month (to avoid the impact of early severe complications); (c) patients with incomplete information on the primary site, surgery, radiation, chemotherapy, pathological grade, cancer stage data, or follow-up data.

Demographic and clinicopathological data such as race, sex, age, year of diagnosis, primary site, histologic type, pathological grade (using a four-grade system of differentiation), surgery, chemotherapy, radiotherapy, tumor size, extent of disease, number of regional nodes examined, TNM stage, and follow-up information were collected. Since the SEER database only provided the stages based on the seventh or earlier edition of the AJCC staging system for patients diagnosed before 2017, we utilized the information from the collaborative stage data collection system in SEER database to re-stratify these patients according to TNM stage, as defined by the AJCC 8th edition, into the following groups: T1 (0–20mm), T2 (21–40mm), T3 (> 40mm), T4 (the celiac axis, superior mesenteric artery, or common hepatic artery involved), N0, N1 (1–3), N2 (> 3), M0, and M1. Some lymph node locations identified as regional nodes by AJCC 6th staging were reclassified as distant nodes by the 8th staging and labeled M1, such as celiac axis and splenic nodes for pancreatic head cancer. Treatment and operative details included the surgical approach (patients who underwent partial pancreatectomy, local or partial pancreatectomy, and duodenectomy with or without partial gastrectomy, total pancreatectomy, total pancreatectomy, and subtotal gastrectomy or duodenectomy, extended pancreatoduodenectomy, pancreatectomy-not otherwise specified (NOS) and surgery-NOS were classified as the surgery group, while others were classified as the non-surgical group), as well as information on adjuvant chemotherapy and radiotherapy. Survival information was retrieved using the phrases “vital status,” “SEER other cause of death classification,” “SEER cause-specific death classification,” and “survival months.” The primary outcomes of interest were OS and CSS. OS was defined as the time from the date of diagnosis to death from any cause, and CSS was defined as the time from the date of diagnosis to death from pancreatic cancer.

### Statistical analysis

Categorical variables are presented as whole numbers or proportions and analyzed using the chi-square test. Survival estimates were generated using the Kaplan–Meier method, and the differences in OS and CSS among groups were evaluated using the log-rank test. To identify potential risk factors for survival, multivariate regression analyses were performed using a Cox proportional hazard model with backward stepwise selection using the Akaike information criterion (AIC) and were reported as the Hazard ratio (HR) with 95% confidence interval (CI). To manage confounding variables associated with prognosis between iIPMN and PDAC patients, a 1:2 nearest neighbor propensity score matching (PSM) with a caliper of 0.02 was performed. Identified factors were included in the nomograms to predict the 1-, 2-, and 3-year OS and CSS rates of iPCN patients who underwent radical surgery. We used restricted cubic splines (RCS) with five knots to flexibly model the association between continuous predictors and death risks. Concordance statistic (C-index) and receiver operating characteristic (ROC) curve were used to evaluate the discriminative ability of the nomogram. A calibration plot was used to evaluate the calibrating ability, and bootstrapping method (1000 repetitions) was used for internal validation of the nomogram. Decision curve analysis (DCA) was performed to compare clinical benefits and usability between the nomogram and TNM staging system. In addition, Kaplan–Meier plots were carried out on the tertiles of patients stratified by the total points predicted by the nomograms using X-tile software (version 3.6.1). All statistical analyses were performed using R software (version 4.1.0) and RStudio (version 2021.09.0 + 351). A two-sided *P* < 0.05 was considered statistically significant.

## Result

### Patient characteristics

**Fig. 1:**
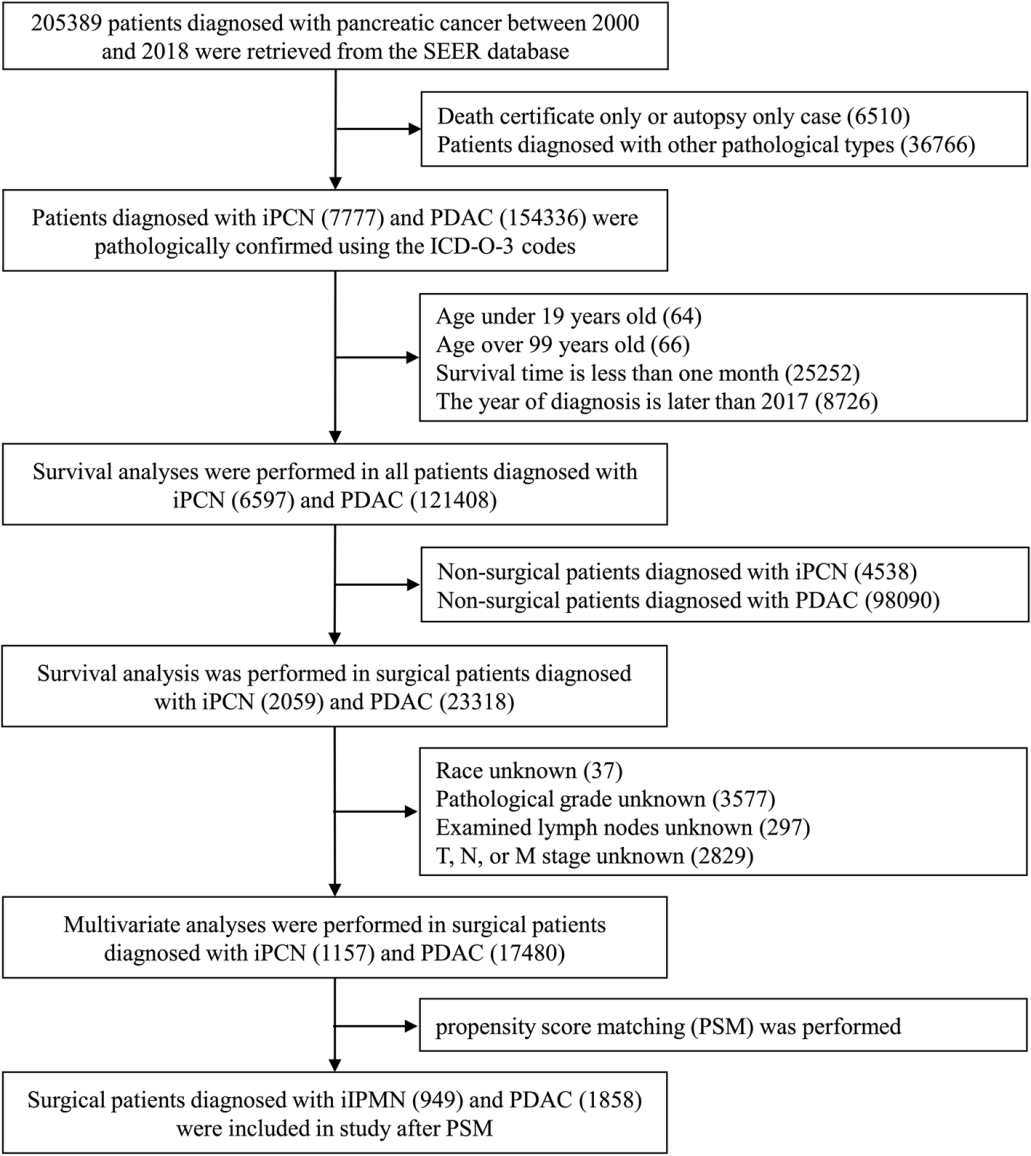
Patient data selection process

The sample selection procedure was illustrated in Fig. 1. The initial query yielded 7777 iPCN patients according to the inclusion criteria, including 6836 iIPMN patients, 504 iMCN patients, 418 iSPN patients, and 19 iSCN patients. In addition, 154,336 PDAC patients were enrolled in this study. The baseline characteristics of patients with iPCN are shown in Table 1. The median patient age at diagnosis was 69 (interquartile range (IQR), 61–77), 68 (IQR, 55–79), 35 (IQR, 24–47), and 71 (IQR, 62–75) years; thus, it can be seen that iSPN mainly occurs in younger women. Among iIPMN patients, the primary lesions were mainly distributed in the pancreatic head, whereas for iMCN and iSPN patients, the lesions were mostly located in the body and tail of the pancreas. Significantly, iIPMN patients had a lower rate of radical surgery compared to other iPCN patients. In total, most neoplasms were advanced, with 10.8% at stage III and 63.1% at stage IV. More than 14 lymph nodes were examined in 32.8% of surgical patients and 8–14 lymph nodes were examined in 26.1% of surgical patients; lymph node metastasis was observed in 34.6% of patients with known lymph node status. Furthermore, the baseline characteristics of surgical patients with iPCN and PDAC are shown in Table S1.

**Table 1:** Demographic and clinicopathological characteristics of pancreatic cancer patients diagnosed with iIPMN, iMCN, iSPN, and iSCN

**Variables**	iIPMN (*N* = 6836) (*N*, %)	iMCN (*N* = 504) (*N*, %)	iSPN (*N* = 418) (*N*, %)	iSCN (*N* = 19) (*N*, %)	SUM (*N* = 7777) (*N*, %)
**Age**					< 0.001
< 56	1007 (14.7)	137 (27.2)	364 (87.1)	2 (10.5)	1510 (19.4)
56–75	3793 (55.5)	194 (38.5)	49 (11.7)	13 (68.4)	4049 (52.1)
> 75	2036 (29.8)	173 (34.3)	5 (1.2)	4 (21.1)	2218 (28.5)
**Sex**					< 0.001
Female	3495 (51.1)	352 (69.8)	355 (84.9)	15 (78.9)	4217 (54.2)
Male	3341 (48.9)	152 (30.2)	63 (15.1)	4 (21.1)	3560 (45.8)
**Race**					< 0.001
White	5562 (81.4)	395 (78.4)	284 (67.9)	15 (78.9)	6256 (80.4)
Black	745 (10.9)	61 (12.1)	78 (18.7)	2 (10.5)	886 (11.4)
Asian-Pacific	510 (7.5)	46 (9.1)	52 (12.4)	2 (10.5)	610 (7.8)
Unknown	19 (0.3)	2 (0.4)	4 (0.1)	0 (0)	29 (0.3)
**Year of diagnosis**					< 0.001
2000–2008	3562 (52.1)	350 (69.4)	78 (18.7)	10 (52.6)	4000 (51.4)
2009–2018	3274 (47.9)	154 (30.6)	340 (81.3)	9 (47.4)	3777 (48.6)
**Primary site**					< 0.001
Head	3002 (43.9)	131 (26.0)	115 (27.5)	6 (31.6)	3254 (41.8)
Body	790 (11.6)	65 (12.9)	70 (16.7)	1 (5.3)	926 (11.9)
Tail	1049 (15.3)	174 (34.5)	160 (38.3)	7 (36.8)	1390 (17.9)
others	1995 (29.2)	134 (26.6)	73 (17.5)	5 (26.3)	2207 (28.4)
**Pathological grade**					< 0.001
I	686 (10.0)	69 (13.7)	66 (15.8)	4 (21.1)	825 (10.6)
II	1212 (17.7)	91 (18.1)	25 (6.0)	1 (5.3)	1329 (17.1)
III–IV	737 (10.8)	49 (9.7)	3 (0.7)	1 (5.3)	790 (10.2)
Unknown	4201 (61.5)	295 (58.5)	324 (77.5)	13 (68.4)	4833 (62.1)
**Regional nodes examined**					< 0.001
0–7	5436 (79.5)	373 (74.0)	225 (53.8)	15 (78.9)	6049 (77.8)
8–14	443 (6.5)	60 (11.9)	100 (23.9)	1 (5.3)	604 (7.8)
> 14	626 (9.2)	51 (10.1)	80 (19.1)	2 (10.5)	759 (9.8)
Unknown	331 (4.8)	20 (4.0)	13 (3.1)	1 (5.3)	365 (4.7)
**T stage**					< 0.001
T1	504 (7.4)	48 (9.5)	43 (10.3)	0 (0.0)	595 (7.7)
T2	1418 (20.7)	67 (13.3)	113 (27.0)	1 (5.3)	1599 (20.6)
T3	1309 (19.1)	201 (40.0)	215 (51.4)	10 (52.6)	1735 (22.3)
T4	967 (14.1)	46 (9.1)	9 (2.2)	3 (15.8)	1025 (13.2)
Tx	3605 (52.7)	142 (28.2)	38 (9.1)	5 (26.3)	3790 (48.7)
**N stage**					< 0.001
N0	1086 (15.9)	190 (37.7)	303 (72.5)	6 (31.6)	1585 (20.4)
N1	545 (8.0)	44 (8.7)	17 (4.1)	2 (10.5)	608 (7.8)
N2	213 (3.1)	15 (3.0)	1 (0.2)	0 (0.0)	229 (2.9)
Nx	4992 (73.0)	255 (50.6)	97 (23.2)	11 (57.9)	5355 (68.9)
**M stage**					< 0.001
M0	2824 (41.3)	354 (70.2)	381 (91.1)	9 (47.4)	3568 (45.9)
M1	3689 (54.0)	106 (21.0)	27 (6.5)	9 (47.4)	3831 (49.3)
Mx	323 (4.7)	44 (8.7)	10 (2.4)	1 (5.3)	378 (4.9)
**TNM stage**					< 0.001
IA	238 (3.5)	31 (6.2)	37 (8.9)	0 (0.0)	306 (3.9)
IB	264 (3.7)	32 (6.3)	88 (21.1)	1 (5.3)	385 (5.0)
IIA	261 (3.8)	90 (17.9)	159 (38.0)	2 (10.5)	512 (6.6)
IIB	334 (4.9)	31 (6.2)	13 (3.1)	2 (10.5)	380 (4.9)
III	611 (8.9)	37 (7.3)	5 (1.2)	1 (5.3)	654 (8.4)
IV	3689 (54.0)	106 (21.0)	27 (6.5)	9 (47.4)	3831 (49.3)
Unknown	1439 (21.1)	177 (35.1)	89 (21.3)	4 (21.1)	1709 (22.0)
**Surgery**					< 0.001
Yes	1653 (23.9)	283 (56.1)	366 (87.5)	10 (52.6)	2312 (29.7)
No/unknown	5183 (76.1)	221 (43.9)	52 (12.5)	9 (47.4)	5465 (70.3)
**Radiotherapy**					< 0.001
Yes	1012 (14.8)	81 (16.1)	8 (1.9)	3 (15.8)	1104 (14.2)
No/unknown	5824 (85.2)	423 (83.9)	410 (98.1)	16 (84.2)	6673 (85.8)
**Chemotherapy**					< 0.001
Yes	3337 (48.8)	155 (30.8)	25 (6.0)	6 (31.6)	3523 (45.3)
No/unknown	3499 (51.2)	349 (69.2)	393 (94.0)	13 (68.4)	4254 (54.7)

*Abbreviations*: *iIPMN* invasive intraductal papillary mucinous neoplasm, *iMCN* invasive mucinous cystic neoplasm, *iSPN* invasive solid pseudopapillary neoplasm, *iSCN* invasive serous cystic neoplasm

### Comparison of survival among iIPMN, iMCN, iSPN, iSCN, and PDAC patients in the SEER database

**Fig. 2:**
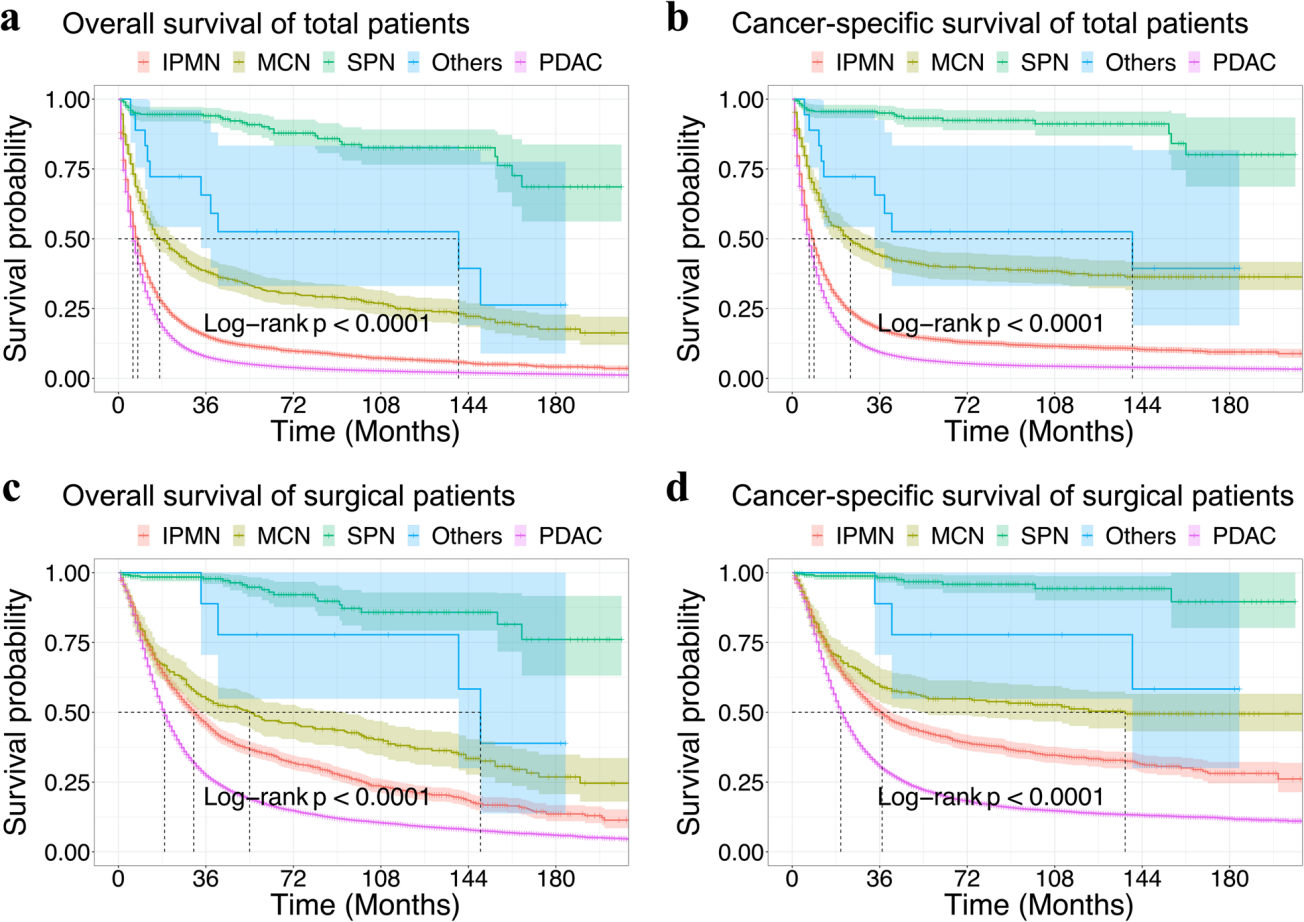
Kaplan–Meier curves demonstrating overall survival (OS) and cancer-specific survival (CSS) in total patients (**a**, **b**) and patients who underwent radical surgery (**c**, **d**) according to histologic type

With regard to OS and CSS, Kaplan–Meier survival analysis was performed in the five pathological groups for the entire population and surgery group (Fig. 2), which showed more favorable outcomes in patients with iSPN and worse outcomes in patients with PDAC. Importantly, pairwise comparison showed that any two of the five pathological groups had a statistically significant difference (except iMCN vs. iSCN, *p* = 0.06 for OS, and *p* = 0.217 for CSS; all other *p* < 0.05, Tables S2, S3, S4 and S5). The 3-year OS and CSS rates in patients who underwent surgery were as follows: 45.7% and 50.1% for iIPMN, 54.8% and 59.3% for iMCN, 97.8% and 98.2% for iSPN, 88.9% and 88.9% for iSCN, and 27.3% and 30.5% for PDAC. In addition, the median OS and CSS times were 6 and 7 months for PDAC patients, compared to a median OS and CSS time of 8 and 9 months for iIPMN patients, and 17 and 24 months for iMCN patients, respectively (*P* < 0.001). By contrast, in the surgery group, the median OS and CSS times were increased to 19 and 20 months for PDAC patients, 31 and 37 months for iIPMN patients, and 54 and 137 months for iMCN patients, respectively (*P* < 0.001).

Multivariate analyses using the Cox proportional hazards model were used to evaluate the impact of known prognostic factors on OS and CSS in the surgery group. Based on the RCS results, the variable of regional nodes examined was categorized in the nomograms (< 8, 8–14, > 14). Similarly, age was stratified as a categorical variable (< 56, 56–75, > 75 years). As shown in Table S6, the types of PDAC and iIPMN, older age at diagnosis, male sex, Black race, head of pancreas, poorly differentiated grade, and more advanced T, N, or M stage were associated with poor outcomes, while the types of iMCN, iSPN, and iSCN, receiving chemotherapy and radiotherapy, diagnosis and treatment in the last decade, and more regional nodes examined were associated with better outcomes.

To investigate the effect of adjuvant therapy on non-distant metastatic patients with different lymph node involvement statuses, multivariate analyses in subgroups of the N stage were performed. The results showed no clinical benefit from chemotherapy or radiotherapy in surgical patients with stage N0 or N1 iIPMN. The OS rate in stage N2 patients with iIPMN was improved by chemotherapy (HR, 1.785; 95% CI, 1.097–2.904; *P* = 0.020 for OS), whereas no significant benefit from radiotherapy was observed in these patients. Owing to the insufficient number of iMCN patients, we only divided them into lymph node-negative and lymph node-positive groups and found that the clinical outcomes of the two groups did not improve with chemotherapy and radiotherapy.

We also explored the disparities in prognostic factors among mucinous types, and the results of multivariate analyses indicated that age, pathologic grade, primary site, T stage, N stage, and regional nodes examined were the main independent risk factors for iIPMN, whereas for iMCN, only age, primary site, and N stage were independent risk factors. The results showed no statistically significant difference in the survival time of between Blacks and Whites among iIPMN and iMCN patients. Meanwhile, we found that patients with iIPMN located at the head of the pancreas were likely to have better prognosis (HR 1.314, *P* = 0.010 for OS; and HR 1.274, *P* = 0.040 for CSS); conversely, iMCN in the body and tail of pancreas had better prognosis (HR 0.542, *P* = 0.048 for OS; and HR 0.453, *P* = 0.023 for CSS).

**Fig. 3:**
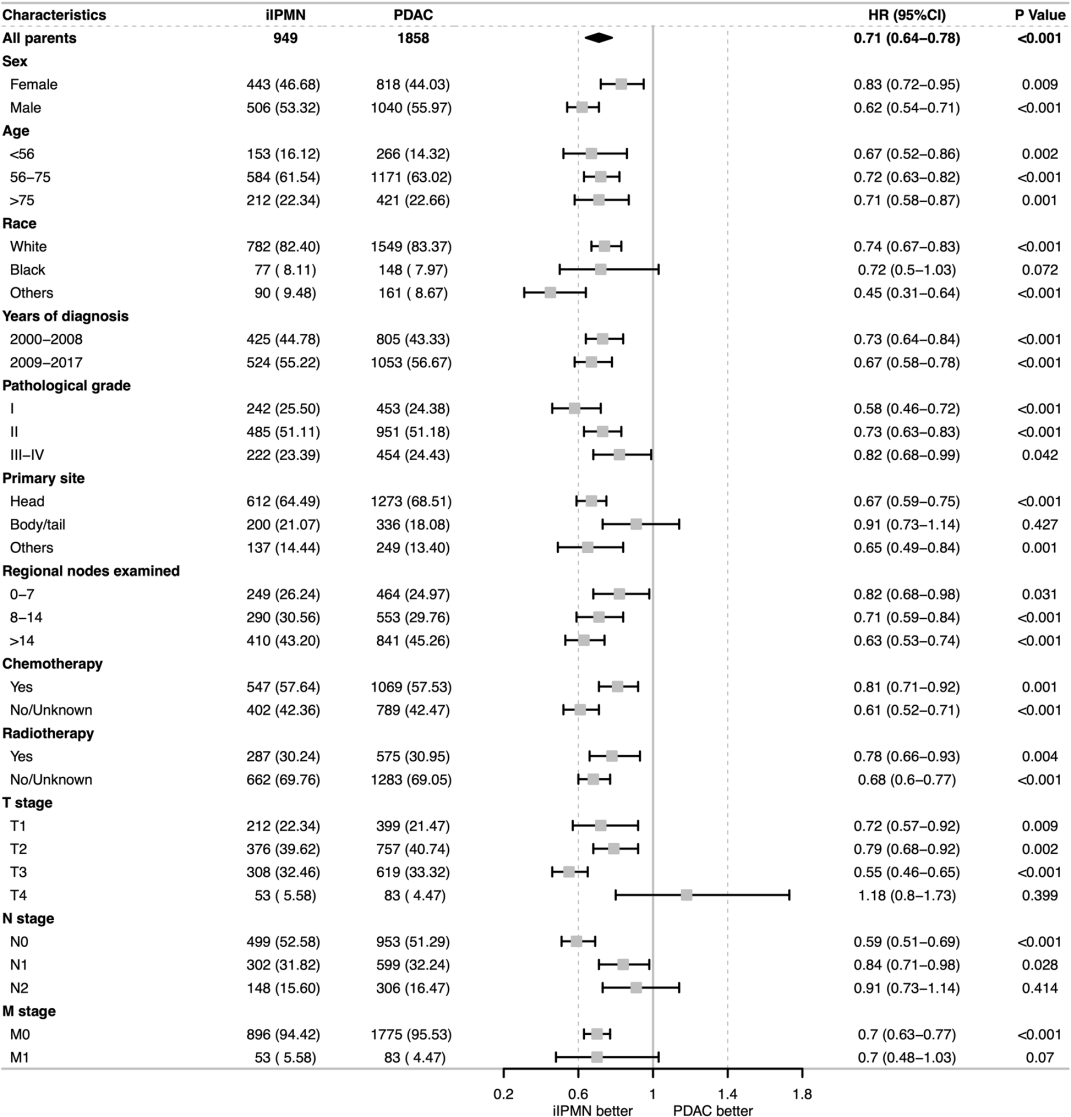
Comparison of cancer-specific survival (CSS) among subgroups of iIPMN and PDAC patients who underwent curative resection after propensity score matching

To further demonstrate the better prognosis of postoperative patients with iIPMN over PDAC, we carried out a PSM between the two groups. Table S7 shows the baseline characteristics of the two groups before and after matching, and the differences in these variables were balanced. A Kaplan–Meier plot was performed in the tendentious matching queue (Fig. S1), which confirmed better prognosis in patients with iIPMN than in those with PDAC [median OS: 29 months vs 21 months (HR 0.747, *P* < 0.001); median CSS: 34 months vs 24 months (HR 0.707, *P* < 0.001)]. However, according to subgroup analysis, there was no statistically significant difference in CSS between postoperative patients with iIPMN and those with PDAC when the patient was Black, and the neoplasm was located in the body and tail of pancreas or staged as T4, N2, or M1 (Fig. 3).

### Construction and validation of nomograms

**Fig. 4:**
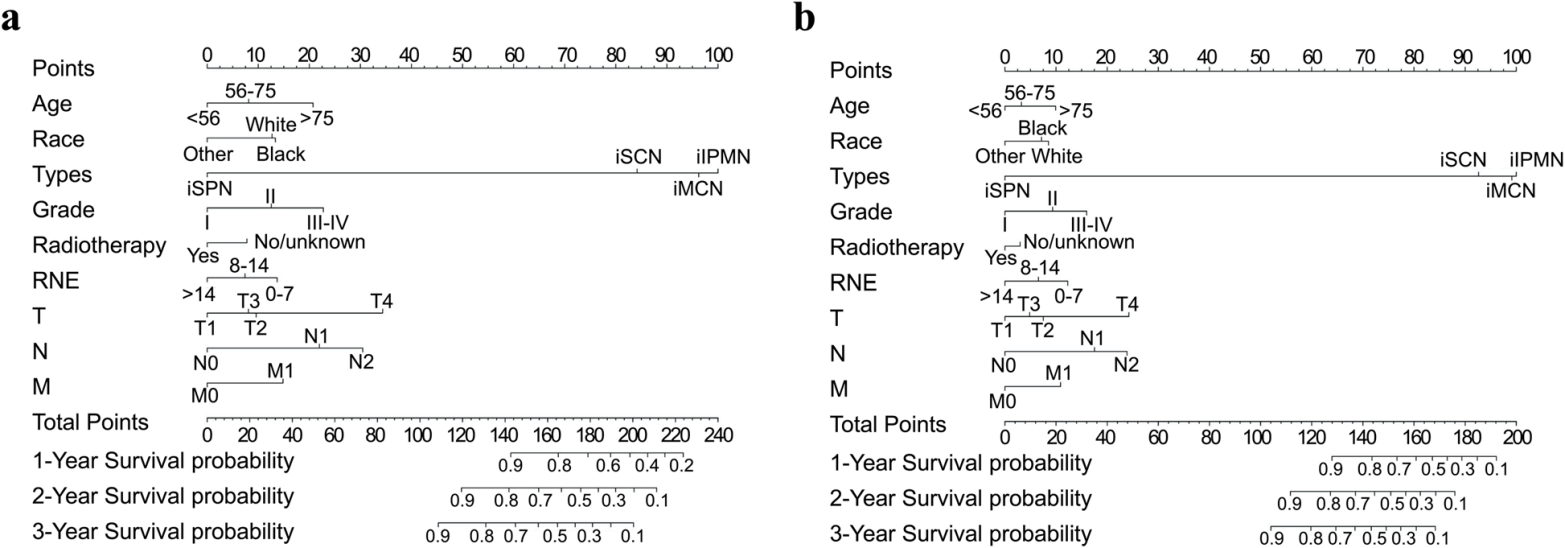
The nomograms to predict overall survival (OS) (**a**) and cancer-specific survival (CSS) (**b**) were performed based on multivariate analyses using the Cox proportional hazard model. Abbreviations: RNE, regional nodes examined

To predict the survival time of patients with iPCN after surgical resection, new prediction models were established based on the Cox proportional hazards regression. Finally, the variables associated with OS and CSS were identified by backward stepwise selection as shown in Table 2. Nomograms to predict the 1-, 2-, and 3-year OS and CSS were created based on the selected variables. Figure 4 illustrates that histologic type contributed the most to survival, while chemotherapy or radiotherapy had the least effect, and lower total points of patients obtained by adding the points for each variable were associated with a more favorable prognosis.

**Table 2:** Multivariate analysis of OS and CSS of iPCN patients who underwent curative resection using the Cox proportional hazards model

**Variables**	OS	*P* value	CSS	*P* value
	HR (95% CI)		HR (95% CI)	
**Age**				
< 56	1 [reference]		1 [reference]	
56–75	1.330 (1.069–1.653)	0.010	1.193 (0.945–1.506)	0.137
> 75	2.050 (1.599–2.627)	< 0.001	1.650 (1.259–2.163)	< 0.001
**Sex**				
Female	1 [reference]		1 [reference]	
Male	1.004 (0.860–1.173)	0.958	0.910 (0.767–1.080)	0.282
**Race**				
White	1 [reference]		1 [reference]	
Black	1.029 (0.788–1.345)	0.832	0.949 (0.699–1.290)	0.740
Others	0.639 (0.482–0.848)	0.002	0.653 (0.476–0.895)	0.008
**Histologic type**				
iIPMN	1 [reference]		1 [reference]	
iMCN	0.836 (0.649–1.077)	0.165	0.901 (0.682–1.191)	0.464
iSPN	0.034 (0.005–0.244)	< 0.001	NA	NA
iSCN	0.628 (0.086–4.562)	0.645	0.751 (0.103–5.479)	0.777
**Pathological grade**				
I	1 [reference]		1 [reference]	
II	1.521 (1.254–1.844)	< 0.001	1.563 (1.254–1.947)	< 0.001
III–IV	2.114 (1.684–2.653)	< 0.001	2.131 (1.655–2.743)	< 0.001
**Year of diagnosis**				
2000–2008	1 [reference]		1 [reference]	
2009–2017	0.827 (0.701–0.976)	< 0.025	0.785 (0.656–0.941)	0.009
**Primary site**				
Head	1 [reference]		1 [reference]	
Body and tail	1.105 (0.911–1.340)	0.312	1.045 (0.842–1.298)	0.690
Others	0.924 (0.684–1.247)	0.603	0.952 (0.685–1.323)	0.769
**Chemotherapy**				
Yes	1 [reference]		1 [reference]	
No/unknown	1.218 (1.011–1.469)	0.038	1.045 (0.851–1.283)	0.674
**Radiotherapy**				
Yes	1 [reference]		1 [reference]	
No/unknown	1.075 (0.889–1.301)	0.455	1.147 (0.934–1.408)	0.190
**Regional nodes examined**				
0–7	1 [reference]		1 [reference]	
8–14	0.829 (0.686–1.003)	0.053	0.789 (0.639–0.973)	0.027
> 14	0.667 (0.548–0.813)	< 0.001	0.603 (0.484–0.751)	< 0.001
**T stage**				
T1	1 [reference]		1 [reference]	
T2	1.388 (1.103–1.747)	0.005	1.458 (1.125–1.890)	0.004
T3	1.309 (1.037–1.651)	0.023	1.266 (0.970–1.653)	0.083
T4	3.176 (2.219–4.547)	< 0.001	3.166 (2.139–4.688)	< 0.001
**N stage**				
N0	1 [reference]		1 [reference]	
N1	2.081 (1.733–2.500)	< 0.001	2.303 (1.881–2.821)	< 0.001
N2	2.822 (2.240–3.554)	< 0.001	3.212 (2.500–4.129)	< 0.001
**M stage**				
M0	1 [reference]		1 [reference]	
M1	1.624 (1.194–2.208)	0.002	1.749 (1.263–2.422)	< 0.001

*Abbreviations*: *OS* overall survival, *CSS* cancer-specific survival, *iPCN* invasive pancreatic cystic neoplasm, *HR* hazard ratio, *iIPMN* invasive intraductal papillary mucinous neoplasm, *iMCN* invasive mucinous cystic neoplasm, *iSPN* invasive solid pseudopapillary neoplasm, *iSCN* invasive serous cystic neoplasm

The discriminative ability of the model was assessed using Kaplan–Meier curves stratified by the tertile of the total points of each patient calculated from the nomograms. As shown in Fig. S2, there were significant differences in the actual OS and CSS of patients between different tertiles, and patients with the lowest points (tertile 1) had noticeably better outcomes (69.6% 3-year OS and 80.1% 3-year CSS) than patients in tertile 2 (26.6% 3-year OS and 53.5% 3-year CSS) and tertile 3 (8.9% 3-year OS and 17.3% 3-year CSS) (*P* < 0.001). In contrast, the approximate median 3-year OS of patients predicted by the model showed good estimates of 70%, 40%, and 15% in tertiles 1, 2, and 3, respectively (*P* < 0.001). Similarly, the median 3-year CSS rates were 75%, 52%, and 21% in tertiles 1, 2, and 3, respectively (*P* < 0.001).

**Fig. 5:**
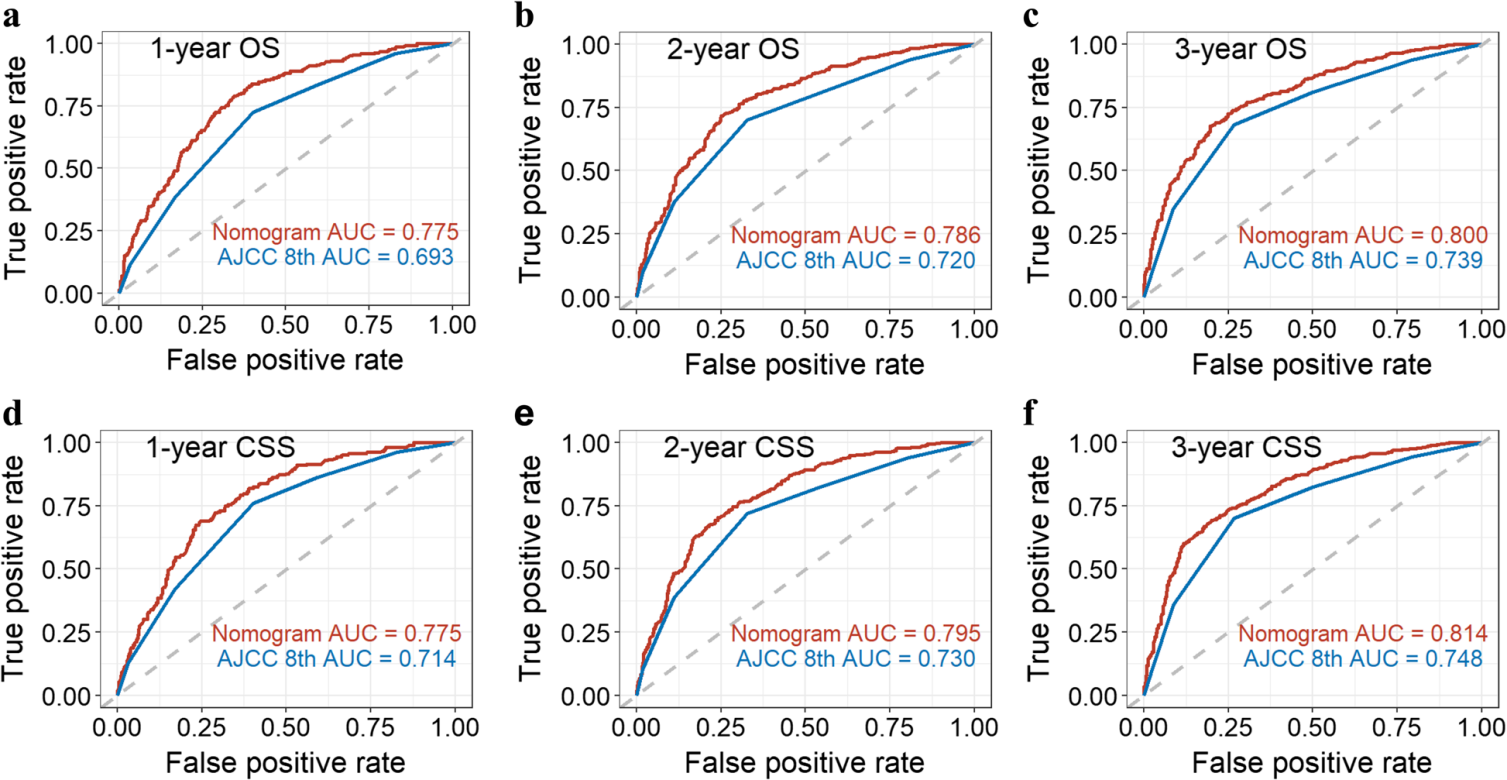
Comparison of ROC curve between the nomogram and the AJCC 8th TNM staging system in prediction of clinical outcomes of iPCN patients who underwent curative resection at 1-, 2-, and 3-year points. **a** One-year OS predicting, **b** 2-year OS predicting, **c** 3-year OS predicting, **d** 1-year CSS predicting, **e** 2-year CSS predicting, and **f** 3-year CSS predicting

**Fig. 6:**
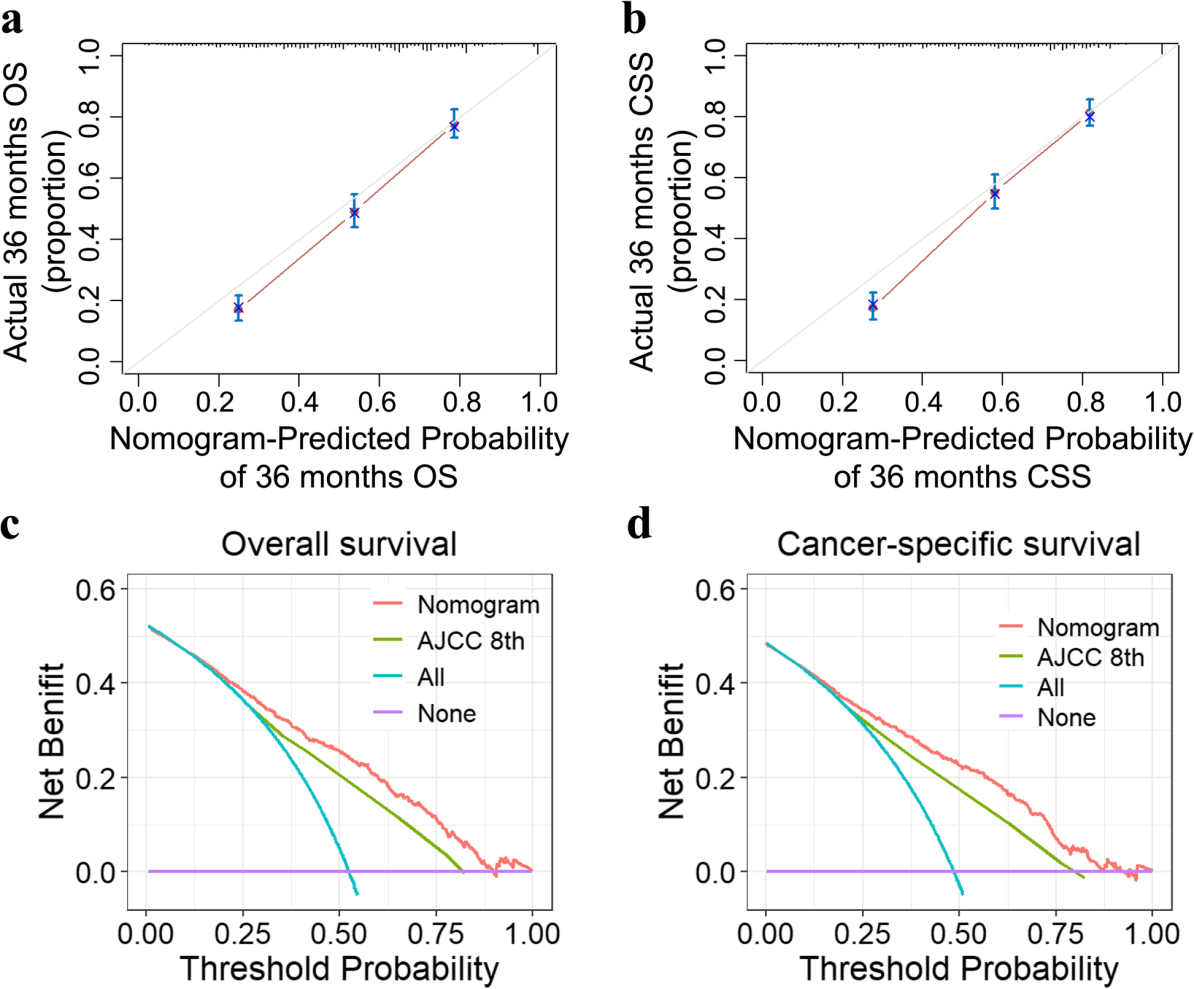
Calibration plot of the nomogram for predicting 3-year overall survival (OS) (**a**) and cancer-specific survival (CSS) (**b**) rates and decision curve analysis (DCA) for the nomogram and the AJCC 8th staging system in prediction of 3-year OS (**c**) and CSS (**d**) rates

To further evaluate the discriminative power of the newly established model, we compared the C-indexes and area under the curve (AUC) values with those of the 8th edition of the TNM staging system. The C-indexes for the new nomogram for the prediction of survival time were higher than those of the TNM staging system (0.725 vs. 0.666 OS and 0.736 vs. 0.683 CSS). The AUC values of the new nomograms that predicted 1-, 2-, and 3-year survival were 0.775, 0.786, and 0.800 (OS) and 0.775, 0.795, and 0.814 (CSS), respectively, whereas the AUC values of the TNM staging system were 0.693, 0.720, and 0.739 (OS) and 0.693, 0.730, and 0.748 (CSS), respectively (all *p* < 0.001) (Fig. 5). Additionally, the comparison of the ROC curve between the two models in the prediction of 5- and 10-year OS and CSS of surgical patients was shown in Fig. S3. The 350-sample bootstrapped calibration plots showed good agreement between the actual and predicted values for the 3-year OS and CSS (Fig. 6a, b). Finally, DCA curves showed that the nomogram has greater clinical benefit in the prediction of 3-year OS and CSS (Fig. 6c, d).

## Discussion

Recently, three important societies and study groups, the International Association of Pancreatology (IAP), the American Gastroenterological Association (AGA), and the European Study Group on Cystic Tumors of the Pancreas, proposed and updated their own guidelines for the diagnosis and management of PCN [6–8]. However, owing to the paucity of available studies, especially comprehensive ones, there has been a lack of consensus on accurate prognostication of outcomes and the optimal modality of surveillance for patients with iPCN in these guidelines. In this study, we identified nearly 8 thousand iPCN patients, including almost all pathological subtypes. We carried out a comprehensive analysis of all iPCN patient data in terms of demographic and clinicopathological characteristics and compared the differences in clinical outcomes between various subtypes. Furthermore, nomograms were created to predict the survival rate of patients with iPCN who underwent curative resection and offered effective information to physicians and patients.

The OS rates for patients with PC have been reported to be very low worldwide [9], and the difference between PDAC and iPCN needs to be validated by large studies [10]. In our survival analysis of total patients, we found that the 3-year OS of iIPMN, iMCN, iSPN, iSCN, and PDAC was 15.2%, 38.2%, 94.0%, 65.7%, and 7.7%, respectively, implying that the prognosis of iPCN is much better than that of PDAC. Likewise, we analyzed patients undergoing radical surgery and found that the prognosis of all types of iPCN was improved and much better than that of PDAC, suggesting that complete surgical resection is also a key treatment modality for patients with iPCN. To further confirm the above findings, we performed multivariate analyses and demonstrated that the risk of death in PDAC increased by 39% compared to that in iIPMN. After adjusting for potential confounders using a propensity score-matched analysis, the results showed that the clinical outcomes of iIPMN were still better than those of PDAC. However, subgroup analysis found that the survival differences were not significant in patients with advanced neoplasms (T4, N2, or M1), which is consistent with several recent studies [11, 12]. Hence, to improve clinical outcomes, long-term monitoring is crucial for the early detection of iIPMN.

Our results confirmed that chemotherapy reduced the risk of death in patients with stage N2 iIPMN, but it had no clinical benefit in lymph node-negative iPCN patients who underwent curative resection. Therefore, we recommend chemotherapy for iIPMN patients with lymph node positivity, which is consistent with previously published studies [13–16]. However, controversy exists on whether adjuvant treatment should be performed in patients without lymph node involvement [17]. The 2018 European guidelines on PCN treatment recommended adjuvant systemic chemotherapy for iIPMN with or without positive lymph node status [8]. Based on our data, adjuvant chemotherapy should not be recommended as conventional therapy for patients with N0 stage, except for concurrent multiple risk factors, such as involvement of the celiac artery, superior mesenteric artery, and common hepatic artery; insufficient lymph nodes detection, and poor differentiation.

There is insufficient data to support radiotherapy for PCN-associated invasive carcinomas. A few studies showed that adjuvant radiotherapy alone or in combination with chemotherapy may be beneficial to node-positive iIPMN patients [14, 18], while no evidence is available to demonstrate the effect of radiotherapy on iMCN patients. Our study confirmed that there was no clinical benefit of radiotherapy in surgical iPCN patients with or without lymph node involvement. Moreover, we found no difference in survival time in iIPMN patients who received chemotherapy combined with radiotherapy or chemotherapy alone, regardless of lymph node involvement status.

In general, the AJCC staging system used for staging PC has a good value for prognostic stratification of the total population; however, the estimation of clinical outcomes for each individual is impractical. Recently, as a more individualized prediction method, nomograms have been shown to provide prognostic information and guide accurate decision-making for treatment [19–21]. Currently, no good predictive model is available for prognostication of patients with PCN [22]. In this study, we created a nomogram to predict the outcomes of surgical patients with iPCN based on the risk factors. Our model showed good discrimination, with an AUC value of 0.800 for predicting 3-year OS and 0.814 for 3-year CSS. We also performed a Kaplan–Meier analysis of the OS and CSS between the high-risk, medium-risk, and low-risk patients, stratified by the total points of each individual calculated by the predictive model. The results showed significant differences among groups, which graphically proved the good discriminative ability. Furthermore, the median predicted 3-year survival probability and the actual 3-year survival probability of each group were similar, which reflected good calibration ability. We also constructed a calibration plot for the prediction of 3-year OS and CSS. The predictions nearly fell along the 45-degree diagonal line, implying that the nomogram was a well-calibrated model. Also, we compared the predictive performance of the new model with the AJCC 8th staging system, and the results of the AUC and DCA showed that the new model has an absolute advantage in terms of discrimination and calibration. Collectively, these data strongly suggest that our proposed predictive model can provide information on the prognosis of iPCN patients undergoing curative resection.

The present study had several limitations. Firstly, some variables are not available in the SEER database, including laboratory test information such as serum CA19-9, which is considered as one of the most effective PC markers, comorbidity information, radiographic findings, and details of intervention, such as the status of the surgical margin and the regimens, sequences, and courses of adjuvant treatment, all of which are important prognostic factors of iPCN based on past experience; hence, the potential effect of these variables in the predictive models could not be evaluated. Secondly, although we collected approximately 20 years’ data on iPCN patients from 18 states across the USA, cases of special types of iPCN were still insufficient due to the extremely low incidence of these cystic cancers. Simultaneously cNET was not listed separately in the SEER database and could not be counted; therefore, partial analysis was limited. Finally, although the proposed nomograms showed better performance by internal validation than the AJCC staging system, more studies using other databases are needed to externally validate our predictive models.

## Conclusions

In conclusion, this large retrospective SEER-based population study showed that the prognosis of patients with iPCN subtypes was significantly better than that of PDAC patients. Several independent prognostic factors were identified to predict the OS and CSS of patients with iPCN who underwent curative resection. Based on these variables, a new survival prediction model was created and presented using nomograms, which demonstrated substantially better discrimination and calibration compared to the 8th AJCC staging system. Future studies are required for optimization of the nomograms through the addition of other important variables, such as laboratory tests, comorbidities, and treatment modalities, and further validation of their predictive value in other databases.

### Supplementary Information

**Additional file 1:** **Figure S1.** Kaplan-Meier curves demonstrating overall survival (OS) (a) and cancer-specific survival (CSS) (b) in iIPMN and PDAC patients who underwent curative resection after propensity score matching.

**Additional file 2:** **Figure S2.** Kaplan-Meier Curves demonstrating overall survival (OS) (a) and cancer-specific survival (CSS) (b) in iPCN patients who underwent curative resection according to tertiles of predicted survival using the nomogram.

**Additional file 3:** **Figure S3.** Comparison of ROC curve between the nomogram and the AJCC 8th TNM staging system in prediction of clinical outcomes of iPCN patients who underwent curative resection at 5- and 10-year point. A: 5-year OS predicting, B: 5-year CSS predicting, C: 10-year OS predicting, D: 10-year CSS predicting.

**Additional file 4:** **Table S1.** Demographic and clinicopathological characteristics of surgical patients diagnosed with iIPMN, iMCN, iSPN, iSCN, and PDAC.

**Additional file 5:** **Table S2.** Pairwise comparisons using log-rank test to assess the overall survival difference between five pathological groups in the entire population.

**Additional file 6:** **Table S3.** Pairwise comparisons using log-rank test to assess the cancer-specific survival difference between five pathological groups in the entire population.

**Additional file 7:** **Table S4.** Pairwise comparisons using log-rank test to assess the overall survival difference between five pathological groups in the surgery patients.

**Additional file 8:** **Table S5.** Pairwise comparisons using log-rank test to assess the cancer-specific survival difference between five pathological groups in the surgery patients.

**Additional file 9:** **Table S6.** Multivariate analysis of OS and CSS of surgical patients diagnosed with iPCN and PDAC using the Cox proportional hazards model.

**Additional file 10:** **Table S7.** Demographic and clinicopathological characteristics of patients diagnosed with iIPMN and PDAC before and after propensity score matching.

## Data Availability

The SEER database is an open-access resource, and the study website https://seer.cancer.gov/ has information on available data and access procedures. The data that support the findings of this study are available from the corresponding author upon reasonable request.
