# Supplementary figures and images for: Clinical characteristics and survival prediction of surgical patients with invasive pancreatic cystic neoplasm: a large retrospective study over two decades
Source: World J Surg Oncol. 2023 Aug 23;21:261. doi: 10.1186/s12957-023-03145-z (PMC10463826; doi:10.1186/s12957-023-03145-z)

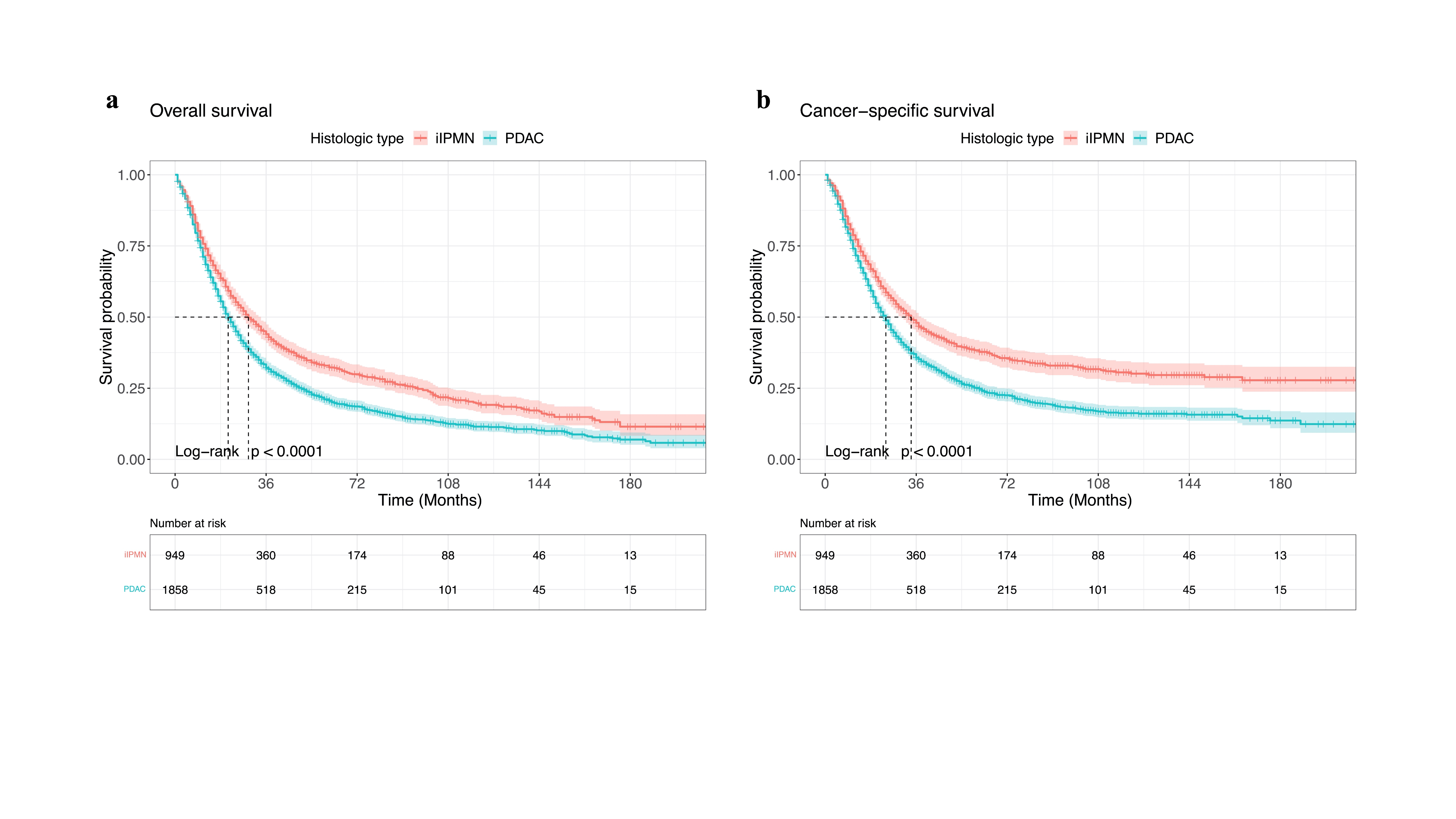

Supplement: Supplementary file 1 — Additional file 1: Figure S1. Kaplan-Meier curves demonstrating overall survival (OS) (a) and cancer-specific survival (CSS) (b) in iIPMN and PDAC patients who underwent curative resection after propensity score matching. [file 12957_2023_3145_MOESM1_ESM.jpg]

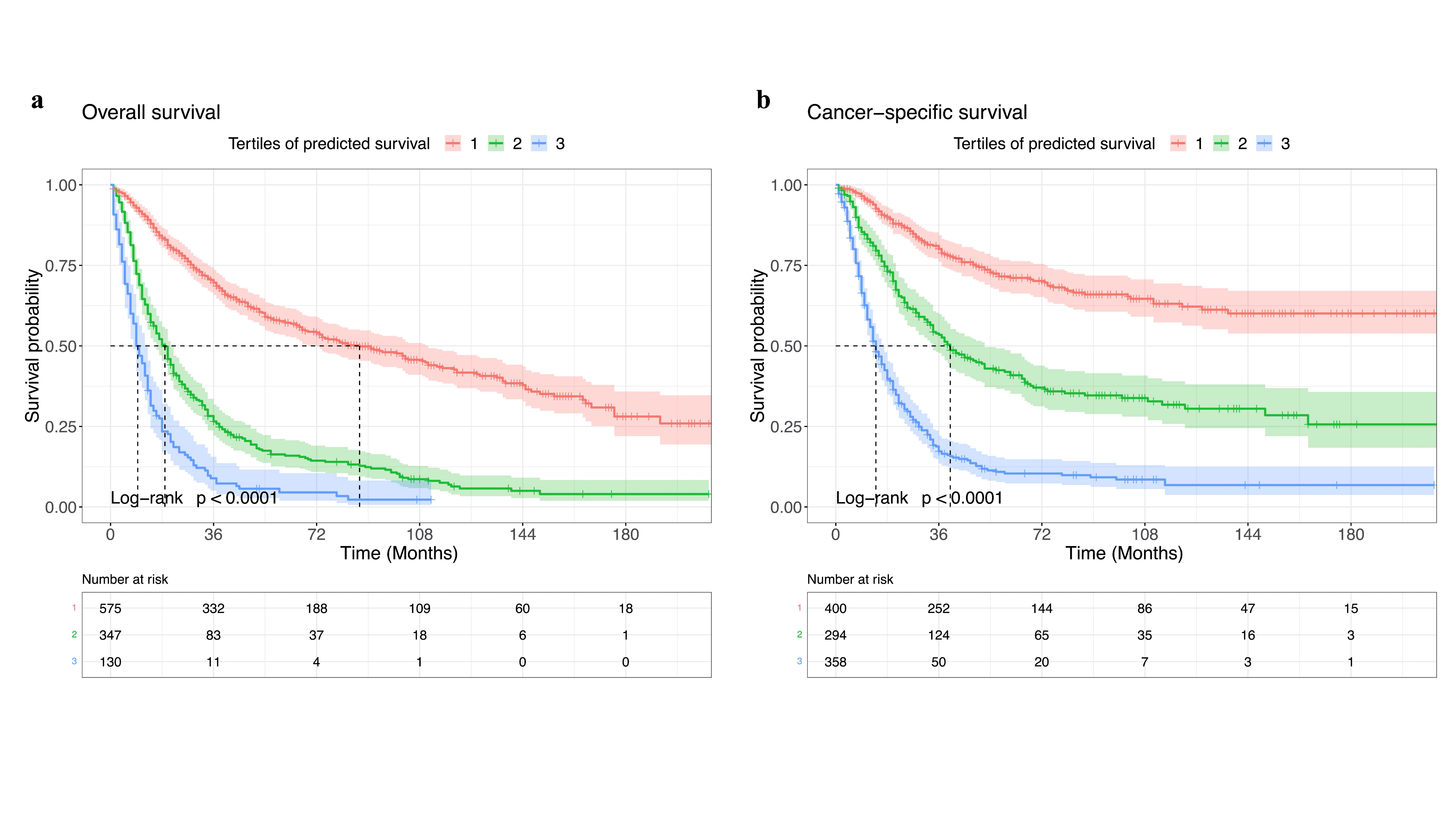

Supplement: Supplementary file 2 — Additional file 2: Figure S2. Kaplan-Meier Curves demonstrating overall survival (OS) (a) and cancer-specific survival (CSS) (b) in iPCN patients who underwent curative resection according to tertiles of predicted survival using the nomogram. [file 12957_2023_3145_MOESM2_ESM.jpg]
