# Supplementary material for: Clinical characteristics and survival prediction of surgical patients with invasive pancreatic cystic neoplasm: a large retrospective study over two decades
Source: World J Surg Oncol. 2023 Aug 23;21:261. doi: 10.1186/s12957-023-03145-z (PMC10463826; doi:10.1186/s12957-023-03145-z)
Supplement: Supplementary file 4 — Additional file 4: Table S1. Demographic and clinicopathological characteristics of surgical patients diagnosed with iIPMN, iMCN, iSPN, iSCN, and PDAC. [file 12957_2023_3145_MOESM4_ESM.docx]

**Table S1**. Demographic and clinicopathological characteristics of surgical patients diagnosed with iIPMN, iMCN, iSPN, iSCN, and PDAC

| **Variables** | iIPMN (N = 1519) (N, %) | iMCN (N = 268) (N, %) | iSPN (N = 263) (N, %) | iSCN (N = 9) (N, %) | PDAC (N = 23318) (N, %) | |
| --- | --- | --- | --- | --- | --- | --- |
| **Age** |  |  |  |  | <0.001 | |
| <56 | 278 (18.3) | 102 (38.1) | 231 (87.8) | 2 (22.2) | 3814 (16.4) |  |
| 56-75 | 916 (60.3) | 117 (43.7) | 32 (12.2) | 6 (66.7) | 14715 (63.1) |  |
| >75 | 325 (21.4) | 49 (18.3) | 0 (0.0) | 1 (11.1) | 4789 (20.5) |  |
| **Sex** |  |  |  |  | <0.001 | |
| Female | 703 (46.3) | 203 (75.7) | 226 (85.9) | 6 (66.7) | 11500 (49.3) | |
| Male | 816 (53.7) | 65 (24.3) | 37 (14.1) | 3 (33.3) | 11818 (50.7) | |
| **Race** |  |  |  |  | <0.001 | |
| White | 1254 (82.7) | 207 (77.5) | 182 (69.2) | 7 (77.8) | 19224 (82.6) | |
| Black | 120 (7.9) | 33 (12.4) | 46 (17.5) | 1 (11.1) | 2295 (9.9) | |
| Asian-Pacific | 142 (9.4) | 27 (10.1) | 35 (13.3) | 1 (11.1) | 1766 (7.6) | |
| Unknown | 3 (0.0) | 1 (0.0) | 0 (0.0) | 0 (0.0) | 33 (0.0) | |
| **Year of diagnosis** |  |  |  |  | <0.001 | |
| 2000-2008 | 754 (49.6) | 188 (70.1) | 56 (21.3) | 5 (55.6) | 9199 (39.5) | |
| 2009-2017 | 765 (50.4) | 80 (29.9) | 207 (78.7) | 4 (44.4) | 14119 (60.5) | |
| **Primary site** |  |  |  |  | <0.001 | |
| Head | 919 (60.5) | 55 (20.5) | 68 (25.9) | 3 (33.3) | 16751 (71.8) | |
| Body | 330 (21.7) | 161 (60.1) | 161 (61.2) | 3 (33.3) | 4004 (17.2) | |
| Tail | 270 (17.8) | 52 (19.4) | 34 (12.9) | 3 (33.3) | 2563 (11.0) | |
| **Pathological grade** |  |  |  |  | <0.001 | |
| I | 311 (20.5) | 55 (20.5) | 47 (17.9) | 4 (44.4) | 2203 (9.4) | |
| II | 582 (38.3) | 75 (28.0) | 21 (8.0) | 0 (0.0) | 10694 (45.9) | |
| III-IV | 258 (17.0) | 41 (15.3) | 0 (0.0) | 0 (0.0) | 7505 (32.2) | |
| Unknown | 368 (24.2) | 97 (36.2) | 195 (74.1) | 5 (55.6) | 2916 (12.5) | |
| **Regional nodes examined** |  |  |  |  | <0.001 | |
| 0-7 | 494 (32.5) | 150 (56.0) | 135 (51.3) | 5 (55.6) | 5930 (25.4) | |
| 8-14 | 417 (27.5) | 58 (21.6) | 70 (26.6) | 1 (11.1) | 6430 (27.6) | |
| >14 | 564 (37.1) | 51 (19.0) | 54 (20.5) | 2 (22.2) | 10381 (44.5) | |
| Unknown | 44 (2.9) | 9 (3.4) | 4 (1.5) | 1 (11.1) | 577 (2.5) | |
| **T stage** |  |  |  |  | <0.001 | |
| T1 | 303 (20.0) | 35 (13.1) | 33 (12.5) | 0 (0.0) | 3328 (14.3) | |
| T2 | 487 (32.1) | 48 (17.9) | 77 (29.3) | 1 (11.1) | 11112 (47.7) | |
| T3 | 444 (29.2) | 140 (52.2) | 138 (52.5) | 6 (66.7) | 4393 (18.8) | |
| T4 | 88 (5.8) | 13 (4.9) | 3 (1.1) | 0 (0.0) | 1475 (6.3) | |
| Tx | 197 (13.0) | 32 (11.9) | 12 (4.6) | 2 (22.2) | 3010 (12.9) | |
| **N stage** |  |  |  |  | <0.001 | |
| N0 | 789 (51.9) | 169 (63.1) | 208 (79.1) | 4 (44.4) | 7688 (33.0) | |
| N1 | 376 (24.8) | 39 (14.6) | 12 (4.6) | 2 (22.2) | 8780 (37.7) | |
| N2 | 197 (13.0) | 15 (5.6) | 1 (0.4) | 0 (0.0) | 4995 (21.4) | |
| Nx | 157 (10.3) | 45 (16.8) | 42 (16.0) | 3 (33.3) | 1855 (8.0) | |
| **M stage** |  |  |  |  | <0.001 | |
| M0 | 1365 (89.9) | 240 (89.6) | 251 (95.4) | 7 (77.8) | 21355 (91.6) | |
| M1 | 126 (8.3) | 21 (7.8) | 10 (3.8) | 1 (11.1) | 1567 (6.7) | |
| Mx | 28 (1.8) | 7 (2.6) | 2 (0.8) | 1 (11.1) | 396 (1.7) | |
| **TNM stage** |  |  |  |  | <0.001 | |
| IA | 215 (14.2) | 29 (10.8) | 28 (10.6) | 0 (0.0) | 1603 (6.9) | |
| IB | 215 (14.2) | 30 (11.2) | 64 (24.3) | 1 (11.1) | 3372 (14.5) | |
| IIA | 225 (14.8) | 82 (30.6) | 103 (39.2) | 2 (22.2) | 1170 (5.0) | |
| IIB | 299 (19.7) | 30 (11.2) | 8 (3.0) | 2 (22.2) | 7031 (30.2) | |
| III | 234 (15.4) | 20 (7.5) | 2 (0.8) | 0 (0.0) | 5603 (24.0) | |
| IV | 126 (8.3) | 21 (7.8) | 10 (3.8) | 1 (11.1) | 1567 (6.7) | |
| Unknown | 205 (13.5) | 56 (20.9) | 48 (18.3) | 3 (33.3) | 2972 (12.7) | |
| **Radiotherapy** |  |  |  |  | <0.001 | |
| Yes | 408 (26.9) | 58 (21.6) | 7 (2.7) | 2 (22.2) | 8096 (34.7) | |
| No/unknown | 1111 (73.1) | 210 (78.4) | 256 (97.3) | 7 (77.8) | 15222 (65.3) | |
| **Chemotherapy** |  |  |  |  | <0.001 | |
| Yes | 786 (51.7) | 97 (36.2) | 14 (5.3) | 3 (33.3) | 15772 (67.6) | |
| No/unknown | 733 (48.3) | 171 (63.8) | 249 (94.7) | 6 (66.7) | 7546 (32.4) | |

Abbreviations: iIPMN, invasive intraductal papillary mucinous neoplasm; iMCN, invasive mucinous cystic neoplasm; iSPN, invasive solid pseudopapillary neoplasm; iSCN, invasive serous cystic neoplasm; PDAC, pancreatic ductal adenocarcinoma
