# Supplementary material for: Clinical characteristics and survival prediction of surgical patients with invasive pancreatic cystic neoplasm: a large retrospective study over two decades
Source: World J Surg Oncol. 2023 Aug 23;21:261. doi: 10.1186/s12957-023-03145-z (PMC10463826; doi:10.1186/s12957-023-03145-z)
Supplement: Supplementary file 8 — Additional file 8: Table S5. Pairwise comparisons using log-rank test to assess the cancer-specific survival difference between five pathological groups in the surgery patients. [file 12957_2023_3145_MOESM8_ESM.docx]

**Table S5.** Pairwise comparisons using log-rank test to assess the cancer-specific survival difference between five pathological groups in the surgery patients

|  | iIPMN | iMCN | iSPN | iSCN |
| --- | --- | --- | --- | --- |
| iMCN | P < 0.001 | - | - | - |
| iSPN | P < 0.001 | P < 0.001 | - | - |
| iSCN | P = 0.046 | P = 0.253 | P = 0.005 | - |
| PDAC | P < 0.001 | P < 0.001 | P < 0.001 | P = 0.002 |
| P value adjustment method: BH | | | | |

Abbreviations: iIPMN, invasive intraductal papillary mucinous neoplasm; iMCN, invasive mucinous cystic neoplasm; iSPN, invasive solid pseudopapillary neoplasm, iSCN, invasive serous cystic neoplasm; PDAC, pancreatic ductal adenocarcinoma; BH, Benjamini–Hochberg method
