# Supplementary material for: Clinical characteristics and survival prediction of surgical patients with invasive pancreatic cystic neoplasm: a large retrospective study over two decades
Source: World J Surg Oncol. 2023 Aug 23;21:261. doi: 10.1186/s12957-023-03145-z (PMC10463826; doi:10.1186/s12957-023-03145-z)
Supplement: Supplementary file 9 — Additional file 9: Table S6. Multivariate analysis of OS and CSS of surgical patients diagnosed with iPCN and PDAC using the Cox proportional hazards model. [file 12957_2023_3145_MOESM9_ESM.docx]

**Table S6.** Multivariate analysis of OS and CSS of surgical patients diagnosed with iPCN and PDAC using the Cox proportional hazards model

| **Variables** | OS |  | CSS |  |
| --- | --- | --- | --- | --- |
|  | HR (95% CI) | P value | HR (95% CI) | P value |
| **Age** |  |  |  |  |
| <56 | 1 [Reference] |  | 1 [Reference] |  |
| 56-75 | 1.143 (1.089-1.200) | <0.001 | 1.081 (1.028-1.137) | 0.002 |
| >75 | 1.431 (1.351-1.515) | <0.001 | 1.303 (1.227-1.384) | <0.001 |
| **Sex** |  |  |  |  |
| Female | 1 [Reference] |  | 1 [Reference] |  |
| Male | 1.071 (1.035-1.107) | <0.001 | 1.046 (1.010-1.084) | 0.012 |
| **Race** |  |  |  |  |
| White | 1 [Reference] |  | 1 [Reference] |  |
| Black | 1.110 (1.048-1.175) | <0.001 | 1.061 (0.998-1.128) | 0.059 |
| Others | 0.993 (0.931-1.058) | 0.823 | 0.991 (0.926-1.060) | 0.792 |
| **Histologic type** |  |  |  |  |
| iIPMN | 1 [Reference] |  | 1 [Reference] |  |
| iMCN | 0.770 (0.613-0.967) | 0.024 | 0.795 (0.619-1.021) | 0.073 |
| iSPN | 0.024 (0.003-0.171) | <0.001 | NA | NA |
| iSCN | 0.531 (0.075-3.785) | 0.528 | 0.661 (0.093-4.711) | 0.680 |
| PDAC | 1.418 (1.306-1.540) | <0.001 | 1.488 (1.359-1.629) | <0.001 |
| **Pathological grade** |  |  |  |  |
| I | 1 [Reference] |  | 1 [Reference] |  |
| II | 1.432 (1.350-1.519) | <0.001 | 1.472 (1.381-1.569) | <0.001 |
| III-IV | 1.906 (1.793-2.026) | <0.001 | 1.977 (1.851-2.112) | <0.001 |
| **Year of diagnosis** |  |  |  |  |
| 2000-2008 | 1 [Reference] |  | 1 [Reference] |  |
| 2009-2017 | 0.852 (0.821-0.883) | <0.001 | 0.833 (0.802-0.866) | <0.001 |
| **Primary site** |  |  |  |  |
| Head | 1 [Reference] |  | 1 [Reference] |  |
| Body and tail | 0.905 (0.863-0.948) | <0.001 | 0.891 (0.847-0.937) | <0.001 |
| Others | 1.019 (0.941-1.102) | 0.645 | 1.040 (0.957-1.130) | 0.354 |
| **Chemotherapy** |  |  |  |  |
| Yes | 1 [Reference] |  | 1 [Reference] |  |
| No/unknown | 1.559 (1.497-1.624) | <0.001 | 1.514 (1.450-1.582) | <0.001 |
| **Radiotherapy** |  |  |  |  |
| Yes | 1 [Reference] |  | 1 [Reference] |  |
| No/unknown | 1.088 (1.046-1.133) | <0.001 | 1.084 (1.040-1.131) | <0.001 |
| **Regional nodes examined** |  |  |  |  |
| 0-7 | 1 [Reference] |  | 1 [Reference] |  |
| 8-14 | 0.858 (0.818-0.899) | <0.001 | 0.842 (0.800-0.885) | <0.001 |
| >14 | 0.714 (0.681-0.749) | <0.001 | 0.695 (0.661-0.731) | <0.001 |
| **T stage** |  |  |  |  |
| T1 | 1 [Reference] |  | 1 [Reference] |  |
| T2 | 1.362 (1.296-1.431) | <0.001 | 1.409 (1.335-1.487) | <0.001 |
| T3 | 1.648 (1.557-1.745) | <0.001 | 1.710 (1.608-1.819) | <0.001 |
| T4 | 2.074 (1.908-2.255) | <0.001 | 2.142 (1.960-2.341) | <0.001 |
| **N stage** |  |  |  |  |
| N0 | 1 [Reference] |  | 1 [Reference] |  |
| N1 | 1.574 (1.511-1.640) | <0.001 | 1.656 (1.585-1.730) | <0.001 |
| N2 | 2.100 (2.000-2.204) | <0.001 | 2.261 (2.147-2.381) | <0.001 |
| **M stage** |  |  |  |  |
| M0 | 1 [Reference] |  | 1 [Reference] |  |
| M1 | 1.965 (1.824-2.117) | <0.001 | 2.010 (1.860-2.171) | <0.001 |

Abbreviations: OS, overall survival; CSS, cancer-specific survival; iPCN, invasive pancreatic cystic neoplasm; PDAC, pancreatic ductal adenocarcinoma; HR, hazard ratio; iIPMN, invasive intraductal papillary mucinous neoplasm; iMCN, invasive mucinous cystic neoplasm; iSPN, invasive solid pseudopapillary neoplasm, iSCN, invasive serous cystic neoplasm
