# Supplementary material for: Clinical characteristics and survival prediction of surgical patients with invasive pancreatic cystic neoplasm: a large retrospective study over two decades
Source: World J Surg Oncol. 2023 Aug 23;21:261. doi: 10.1186/s12957-023-03145-z (PMC10463826; doi:10.1186/s12957-023-03145-z)
Supplement: Supplementary file 10 — Additional file 10: Table S7. Demographic and clinicopathological characteristics of patients diagnosed with iIPMN and PDAC before and after propensity score matching. [file 12957_2023_3145_MOESM10_ESM.docx]

**Table S7.** Demographic and clinicopathological characteristics of patients diagnosed with iIPMN and PDAC before and after propensity score matching.

| **Variables** | before propensity score matching | | | after propensity score matching | | |
| --- | --- | --- | --- | --- | --- | --- |
|  | PDAC (n=17480) | iIPMN (n=966) | P value | PDAC (n=1858) | iIPMN (n=949) | P value |
| **Age (%)** |  |  | 0.549 |  |  | 0.444 |
| <56 | 2726 (15.6) | 156 (16.1) |  | 266 (14.3) | 153 (16.1) |  |
| 56-75 | 11050 (63.2) | 594 (61.5) |  | 1171 (63.0) | 584 (61.5) |  |
| >75 | 3704 (21.2) | 216 (22.4) |  | 421 (22.7) | 212 (22.3) |  |
| **Sex (%)** |  |  | 0.075 |  |  | 0.194 |
| Male | 8832 (50.5) | 517 (53.5) |  | 1040 (56.0) | 506 (53.3) |  |
| **Race (%)** |  |  | 0.032 |  |  | 0.756 |
| White | 14391 (82.3) | 794 (82.2) |  | 1549 (83.4) | 782 (82.4) |  |
| Black | 1716 (9.8) | 78 (8.1) |  | 148 (8.0) | 77 (8.1) |  |
| Others | 1373 (7.9) | 94 (9.7) |  | 161 (8.7) | 90 (9.5) |  |
| **Year of diagnosis (%)** |  |  | <0.001 |  |  | 0.486 |
| 2009-2017 (%) | 10949 (62.6) | 530 (54.9) |  | 1053 (56.7) | 524 (55.2) |  |
| **Primary site (%)** |  |  | <0.001 |  |  | 0.084 |
| Head | 12831 (73.4) | 618 (64.0) |  | 1273 (68.5) | 612 (64.5) |  |
| Body/tail | 2943 (16.8) | 201 (20.8) |  | 336 (18.1) | 200 (21.1) |  |
| Others | 1706 (9.8) | 147 (15.2) |  | 249 (13.4) | 137 (14.4) |  |
| **Chemotherapy (%)** |  |  | <0.001 |  |  | 0.990 |
| No/unknown | 5632 (32.2) | 415 (43.0) |  | 789 (42.5) | 402 (42.4) |  |
| **Radiotherapy (%)** |  |  | 0.008 |  |  | 0.734 |
| No/unknown | 11517 (65.9) | 677 (70.1) |  | 1283 (69.1) | 662 (69.8) |  |
| **Pathological grade (%)** |  |  | <0.001 |  |  | 0.740 |
| I | 1822 (10.4) | 259 (26.8) |  | 453 (24.4) | 242 (25.5) |  |
| II | 9273 (53.0) | 485 (50.2) |  | 951 (51.2) | 485 (51.1) |  |
| III-IV | 6385 (36.5) | 222 (23.0) |  | 454 (24.4) | 222 (23.4) |  |
| **Regional nodes examined (%)** |  |  | <0.001 |  |  | 0.569 |
| 0-7 | 3532 (20.2) | 252 (26.1) |  | 464 (25.0) | 249 (26.2) |  |
| 8-14 | 5319 (30.4) | 293 (30.3) |  | 553 (29.8) | 290 (30.6) |  |
| >14 | 8629 (49.4) | 421 (43.6) |  | 841 (45.3) | 410 (43.2) |  |
| **T stage (%)** |  |  | <0.001 |  |  | 0.537 |
| T1 | 2917 (16.7) | 214 (22.2) |  | 399 (21.5) | 212 (22.3) |  |
| T2 | 9783 (56.0) | 376 (38.9) |  | 757 (40.7) | 376 (39.6) |  |
| T3 | 3862 (22.1) | 322 (33.3) |  | 619 (33.3) | 308 (32.5) |  |
| T4 | 918 (5.3) | 54 (5.6) |  | 83 (4.5) | 53 (5.6) |  |
| **N stage (%)** |  |  | <0.001 |  |  | 0.766 |
| N0 | 5953 (34.1) | 516 (53.4) |  | 953 (51.3) | 499 (52.6) |  |
| N1 | 7268 (41.6) | 302 (31.3) |  | 599 (32.2) | 302 (31.8) |  |
| N2 | 4259 (24.4) | 148 (15.3) |  | 306 (16.5) | 148 (15.6) |  |
| **M stage (%)** |  |  | 0.425 |  |  | 0.226 |
| M1 | 850 (4.9) | 53 (5.5) |  | 83 (4.5) | 53 (5.6) |  |

Abbreviations: PDAC, pancreatic ductal adenocarcinoma; iIPMN, invasive intraductal papillary mucinous neoplasm
